# Comparison of measures for haplotype similarity

**DOI:** 10.1186/1753-6561-1-s1-s128

**Published:** 2007-12-18

**Authors:** Vivien Marquard, Lars Beckmann, Justo L Bermejo, Christine Fischer, Jenny Chang-Claude

**Affiliations:** 1 Cancer Epidemiology, German Cancer Research Center DKFZ, Im Neuenheimer Feld 280, 69120 Heidelberg, Germany; 2 Molecular Genetic Epidemiology, German Cancer Research Center DKFZ, Im Neuenheimer Feld 280, 69120 Heidelberg, Germany; 3 Institute of Human Genetics, University of Heidelberg, Im Neuenheimer Feld 366, 69120 Heidelberg, Germany

## Abstract

Measuring the association of haplotype similarities with phenotype similarities has been used to develop statistical tests of genetic association. Previously, we applied the general approach of Mantel statistics to correlate genetic and phenotype similarity, where genetic similarity was defined by the number of intervals flanked by markers identical by state for pairs of haplotypes. Here we investigated in the case-control study design the effect on power of the Mantel statistics for five different measures of genetic similarity based on haplotypes: 1) the number of shared intervals, 2) the physical length of the shared intervals, 3) the genetic length of the shared intervals in centimorgans, 4) the genetic length of the shared intervals in linkage disequilibrium units (LDU) and 5) Yu's measure that attaches more weight to the sharing of rare than common alleles. With prior knowledge of the answers of Genetic Analysis Workshop 15 Problem 3, we analyzed the simulated data sets in two genomic regions surrounding the disease loci on chromosomes 6 and 18. For the dense map on chromosome 6, all methods showed a very high power of comparable magnitude. For chromosome 18, we observed a power between 19% and 99% at the pointwise 5% significance level using 1000 cases and 1000 controls for all methods except Yu's measure. While it yielded a much lower power, Yu's measure had 80% power around the disease locus.

## Background

The potential value of haplotypes in the mapping of complex traits has attracted widespread interest. A convenient approach to incorporating haplotype information is the search for shared chromosomal segments. Haplotype-sharing approaches are based on the assumption that, in the vicinity of a predisposing mutation, haplotypes carrying this mutation are more similar than haplotypes without the mutation. The expectation is that the case haplotypes share significantly longer stretches of DNA identically by descent (IBD) around the mutation. Thus, the first proposed measure of similarity between haplotypes for gene mapping was the number of intervals flanked by the same marker alleles, i.e., by markers identical by state (IBS) [1]. However, this approach does not take into account marker spacing and linkage disequilibrium (LD). This study investigated whether alternative haplotype similarity measures improve the power of haplotype-sharing analysis. We weighted the intervals by their physical length, and by their genetic length measured in centimorgans and in linkage disequilibrium units (LDUs). Furthermore, we studied an approach that gives more weights to the sharing for rare marker alleles than for common ones.

To analyze the dependence of power on the different similarity measures, we used a previously developed Mantel statistic to correlate genetic and phenotypic similarity [2]. We used the simulated data sets of Genetic Analysis Workshop 15 (GAW15) Problem 3 in two genomic regions for a population-based case-control scenario.

## Methods

### Simulated data sets

From the simulated rheumatoid arthritis data set consisting of 1500 families with two affected children and 2000 unrelated controls (Problem 3), the first affected child from each of the first 1000 families was chosen to constitute the case group. For each replication the cases were matched by sex with 1000 controls. With prior knowledge of the disease-causing loci, 21 SNPs (3427 to 3447) including Locus C of the high-density scan of chromosome 6 were extracted. In addition, 20 SNPs (260 to 279) from chromosome 18 around Locus E were chosen.

Because of the known strong effect of Locus C, smaller samples each consisting of 50 or 100 cases and controls were used for the analysis of chromosome 6. Females and males were analyzed separately because of the known gender-specific interaction between Locus C and the Disease Locus DR. Data on chromosome 18 was analyzed using samples of 500 or 1000 cases and controls for both sexes combined. The haplotypes used for analysis were provided by GAW15.

### Measures of haplotype similarity *L*_*ij*_(*x*)

We applied four different measures of haplotype similarity based on the number of shared intervals, i.e., number of intervals surrounding a marker that are flanked by markers with the same alleles (IBS). Modified versions of these four measures were also employed to take into account the sharing of a single marker and the unobserved regions between the examined markers beyond the shared region. The first and common measure *N *counts the number of shared intervals in the vicinity of a specific marker. The modified version of this measure, *N*+, corresponds to *N *+ 1. For the next three measures, *N *was weighted with the physical, KB, or genetic length, CM, or LDU, between the first and the last shared markers in (kilo)base pairs, centimorgans, or LDUs, respectively. LDUs were introduced by Morton et al. as a genetic distance based on the observed haplotype frequencies [3]. Maniatis et al. showed that the use of LDUs might improve the power of single-point linkage analysis and the power to identify disease-causing variants [4]. The software LDMAP [4] has been used to determine LDUs of the chosen markers. For the modified versions, KB+, CM+, and LDU+, the half of the distance both before the first shared marker and after the last shared marker were added. When either the first or the last of all investigated markers was involved, then half of the distance to the second and to the penultimate marker was used as a proxy, respectively. We also studied the measure proposed by Yu et al. [5], which gives greater weights to the sharing for rare marker alleles than to that for common alleles. The weights are determined as the probability of particular alleles at the specific marker, conditional on the surrounding alleles and are estimated from the allele frequencies of control haplotypes.

### Exploratory analysis

Kruskal's nonmetric multidimensional scaling (MDS) was used to explore the resemblance among the different similarity measures [6]. MDS performs a minimizing algorithm based on the stress value, a least square estimator that assesses the dimensionality of the data using the observed and estimated distances. The smaller the stress value, the better the fit, with stress values between 0 and 2.5 indicating an excellent goodness of fit. After dimensionality assessment, a graphical representation of the data permitted the investigation of resemblance among similarity measures.

### Mantel statistics using haplotype sharing and power analysis

The haplotype-based Mantel statistic correlates the haplotype similarity *L*_*ij *_for every marker *x*, where *i *and *j *are two haplotypes, and the phenotypic similarity $Y_{s_{i}s_{j}}$ from two individuals *s*_*i *_and *s*_*j *_corresponding to the haplotypes *i *and *j *[2]. The phenotypic similarity $Y_{s_{i}s_{j}}$ is defined as the mean corrected product $Y_{s_{i}s_{j}}=(Y_{s_{i}}-\mu )(Y_{s_{j}}-\mu )$, where *μ *denotes the expectation of the phenotype in the sample, i.e., *μ *= 0.5, while a case was coded as 1 and a control as 0. Thus, the defined statistic is the sum of the cross products of *L*_*ij*_(*x*) and $Y_{s_{i}s_{j}}$:

$$M(x)=\sum_{i<j}L_{ij}(x)Y_{s_{i}s_{j}}.$$

Statistical significance was assessed via a Monte Carlo permutation. The empirical null distribution, i.e., the distribution in which the genetic and the phenotypic similarity were independently distributed, was estimated by permuting the phenotype 1000 times while keeping together the two haplotypes derived from an individual. The empirical *p*-value was derived by comparing the observed statistic against the empirical distribution.

The different haplotype similarity measures were calculated for each replication *r *= 1,..., 100 for both chromosomes and employed to investigate the power of the Mantel statistics to map the disease locus. The power was estimated as the number of replications with a significant test result (*p*-value less than *α *= 0.05) divided by 100, the total number of replications.

The data management, the calculation of the similarity measures as well as the Mantel statistics were performed in the R programming language.

## Results

**Figure 1:**
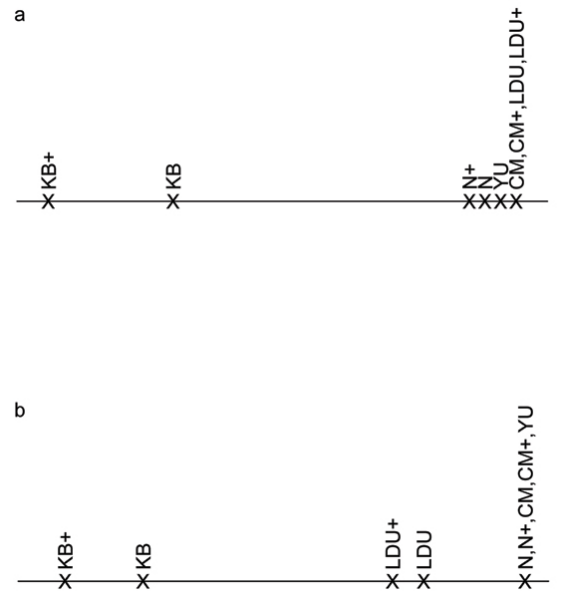
**Results from MDS analyses**. Results from MDS analyses for chromosome 6 (a) and chromosome 18 (b). The distances among the abbreviations on the axis reflect the resemblance among the similarity measures.

**Figure 2:**
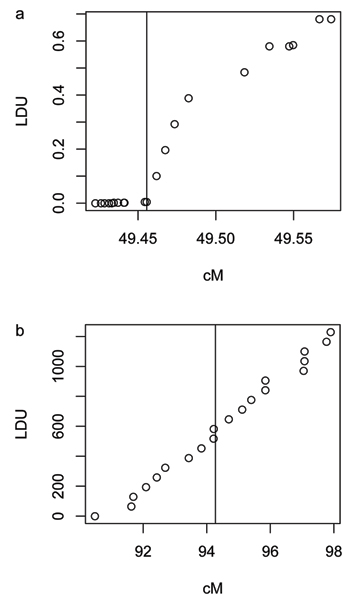
**Relationship between the genetic distance measures**. Comparison of the genetic distances in LDU with the genetic distances in centimorgans for chromosome 6 (a) and chromosome 18 (b). Vertical line denotes the location of the disease locus.

The exploratory analysis using MDS yielded values of stress = 0.01 for chromosome 6 and stress = 0.80 for chromosome 18, both indicating an excellent goodness of fit. Therefore, a one-dimensional representation was sufficient to describe the resemblance between the similarity measures (Fig. 1a, b). For chromosome 6, the results showed a clear difference between physical distances (in particular KB and KB+) and the other similarity measures. The number of shared intervals (N, N+) and YU were slightly different from genetic distances (CM, CM+, LDU, and LDU+), which were undistinguishable (Fig. 1a). For chromosome 18, three clusters of similarity measures were identified by MDS: i) KB, KB+, ii) LDU, LDU+, and iii) the remaining measures N, N+, CM, CM+, and YU (Fig. 1b). The relationship between the genetic distances measured in centimorgans and LDUs is shown in Figure 2. On chromosome 6, the genetic distances in centimorgans among the first ten markers were small, but they were null when measured in LDUs, indicating complete LD in this region (Fig. 2a). On chromosome 18, LDUs and centimorgan distances were approximately linearly correlated (Fig. 2b). The markers covered a larger region on chromosome 18 (4000 kb) than on chromosome 6 (300 kb), but gaps can be observed on the genetic scales for both chromosomal regions.

**Figure 3:**
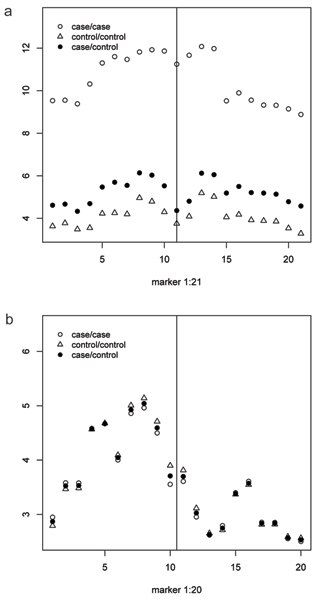
**Mean sharing**. Case-case, case-control, and control-control haplotype pairs, mean sharing for similarity measure *N *for every examined marker on chromosome 6 (a) and chromosome 18 (b). Vertical lines denote the location of the disease locus.

The measure of haplotype similarity *N *between haplotypes *i *and *j *at marker position *x*, *L*_*ij*_(*x*), was calculated for case-case, case-control, and control-control haplotype pairs. Figure 3a displays the mean sharing for these groups at every marker position *x *on chromosome 6. For each similarity measures employed, the mean sharing among case-case pairs was larger than for case-control or control-control pairs, indicating that cases share longer stretches of haplotypes than controls in the vicinity of the susceptibility gene. However, for chromosome 18 this pattern was not observed (Fig. 3b).

**Figure 4:**
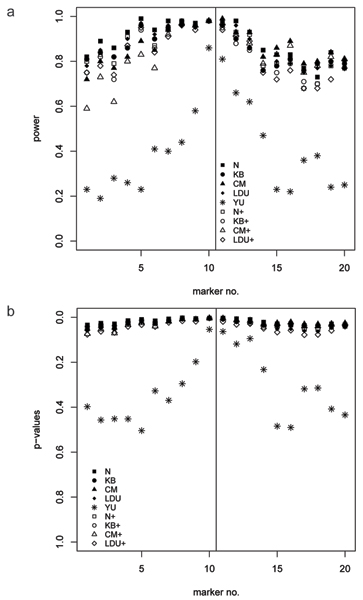
**Power and *p*-values**. Comparison of power (a) and *p*-values (b) for the nine haplotype similarity measures for each examined marker on chromosome 18 with a sample size of 1000. Vertical lines denote the location of the disease locus.

For males, the power to identify Locus C on chromosome 6 was 100% for all measures in both investigated sample sizes. For females, the power was 100% for all measures except LDU and LDU+ using sample sizes of 50 and 100 (data not shown). In contrast, the power to detect Locus E on chromosome 18 did not exceed 43% using a sample size of 500, and Yu's measure had a lower power than the other similarity measures for all examined markers (data not shown). However, with a sample size of 1000, all measures, except that of YU, yielded a high power of around 98% at the markers around the disease locus, but showed different decay of power at distal markers. Whereas Yu's measure showed a power of 80% around the disease locus (~15% less than the other similarity measures), it had a substantially low power of around 20% at the distal markers (40–60% less than the other measures) (Table 1 and Fig. 4a). The pronounced peak of the power using Yu's measure corresponded with low *p*-values (Fig. 4b).

**Table 1:** Power

Marker no.^a^	*N*	N+	KB	KB+	CM	CM+	LDU	LDU+	YU
260	0.82	0.8	0.81	0.75	0.72	0.59	0.78	0.75	0.23
261	0.89	0.83	0.84	0.82	0.8	0.73	0.85	0.78	0.19
262	0.86	0.79	0.82	0.72	0.77	0.62	0.82	0.74	0.28
263	0.93	0.91	0.86	0.87	0.82	0.8	0.9	0.87	0.26
264	0.99	0.99	0.96	0.94	0.89	0.83	0.97	0.95	0.23
265	0.94	0.87	0.9	0.86	0.85	0.77	0.92	0.84	0.41
266	0.98	0.95	0.95	0.94	0.94	0.92	0.96	0.91	0.4
267	0.98	0.98	0.97	0.98	0.97	0.97	0.96	0.98	0.44
268	0.97	0.96	0.96	0.96	0.96	0.96	0.95	0.94	0.58
**269**	**0.98**	**0.98**	**0.98**	**0.98**	**0.98**	**0.98**	**0.98**	**0.98**	**0.86**
**270**	**0.98**	**0.97**	**0.96**	**0.95**	**0.99**	**0.97**	**0.96**	**0.94**	**0.81**
271	0.98	0.93	0.9	0.88	0.93	0.92	0.96	0.91	0.66
272	0.93	0.92	0.86	0.85	0.93	0.88	0.9	0.89	0.62
273	0.85	0.78	0.76	0.76	0.82	0.79	0.82	0.75	0.47
274	0.83	0.79	0.78	0.75	0.86	0.83	0.8	0.72	0.23
275	0.83	0.81	0.81	0.79	0.89	0.87	0.79	0.76	0.22
276	0.77	0.68	0.76	0.71	0.79	0.75	0.75	0.68	0.36
277	0.73	0.7	0.79	0.7	0.8	0.78	0.77	0.68	0.38
278	0.84	0.78	0.8	0.8	0.84	0.82	0.78	0.72	0.24
279	0.8	0.77	0.79	0.79	0.81	0.81	0.77	0.77	0.25

^a^Power comparison for the nine haplotype similarity measures for each examined marker on chromosome 18, where markers 269 and 270 are the flanking markers of the disease locus.

## Discussion

MDS results indicated that for both regions the similarity measures based on physical distances, KB and KB+, are clearly distinguishable from the other investigated measures. The genetic measures CM(+) and LDU(+) were not found to be different for chromosome 6 with dense marker spacing. For chromosome 18 with broader marker spacing, the genetic measures based on LDUs were clearly distinguished from the other measures, although they were correlated with CM(+) (Fig. 2b). LDUs and centimorgans are different measures of genetic distance between markers. LDUs were estimated from the sample of 500 cases and 500 controls and the distances in centimorgans were provided by Genetic Analysis Workshop 15 (GAW15). Estimates of centimorgans and LDU on a very fine scale require sophisticated methods with assumptions about the coalescence process and may therefore be biased and highly variable. Indeed, we observed that the non-linear relationship of LDUs and centimorgans (Fig. 2a) changed to a linear correlation, when 21 markers from a broader region (mean distances between markers 24 kb instead of 15 kb) around the disease locus on chromosome 6 were considered (data not shown). The resemblance among the different similarity measures based on the MDS analyses was not reflected in the results of the power analysis.

## Conclusion

Based on the results for chromosome 18 using a sample size of 1000, the alternative haplotype similarity measures, except YU, are not superior to the simplest measure, defined as the number of intervals flanked by markers IBS. The observations for Yu's measure suggest that incorporation of marker allele frequencies in the similarity measures may improve the fine mapping properties of haplotype sharing methods. Future work will consider a broader spectrum of genomic regions, including different patterns of physical and genetic marker spacing.

## Competing interests

The author(s) declare that they have no competing interests.
